# Longitudinal and circumferential strain assesment of left and right ventricles in acute myocardial infarction superimposed with microemboli

**DOI:** 10.1186/1532-429X-16-S1-P345

**Published:** 2014-01-16

**Authors:** Mohammed S Suhail, Robert Jablonowski, Loi Do, Mark W Wilson, Maythem Saeed

**Affiliations:** 1 Department of Radiology and Biomedical Imaging, School of Medicine, University of California San Francisco, San Francisco, California, USA

## Background

Microvascular obstruction is a major problem after reperfusion therapy of AMI. A recent study reported that longitudinal and circumferential strain after reperfusion therapy is an excellent predictor of left ventricular (LV) dysfunction in patients with anterior wall AMI [1,2]. In the current MRI investigation, we adapted a method routinely used in echocardiography for measuring LV, RV interventricular walls longitudinal strain [3]. Furthermore, we developed a well-controlled AMI superimposed with microemboli model to study LV and RV strain changes and compared it with animals subjected to LAD microembolization or occlusion/reperfusion.

## Methods

Pigs (n = 24) were subjected to either 90 min LAD occlusion plus delivery of 32 mm^3^microemboli then reperfusion (n = 8), LAD occlusion/reperfusion (n = 8) or microembolization (n = 8). Eight animals served as controls. Three days after interventions, 4-chamber view cine MRI (TR/TE/FA = 3.5 ms/1.75 ms/70) was performed in long-axis view for longitudinal strain and 2-chamber view tagged MRI (TR/TE/FA = 35/6.1 ms/25) for circumferential strain. Phasic and peak circumferential strain was analyzed using HARP. For viability imaging, delayed Gd-DTPA enhanced IR-GRE sequence was used (TR/TE/FA = 5 ms/2 ms/15°). Nonparametric Student and Dunn's multiple comparison tests were used.

## Results

**Figure 1:**
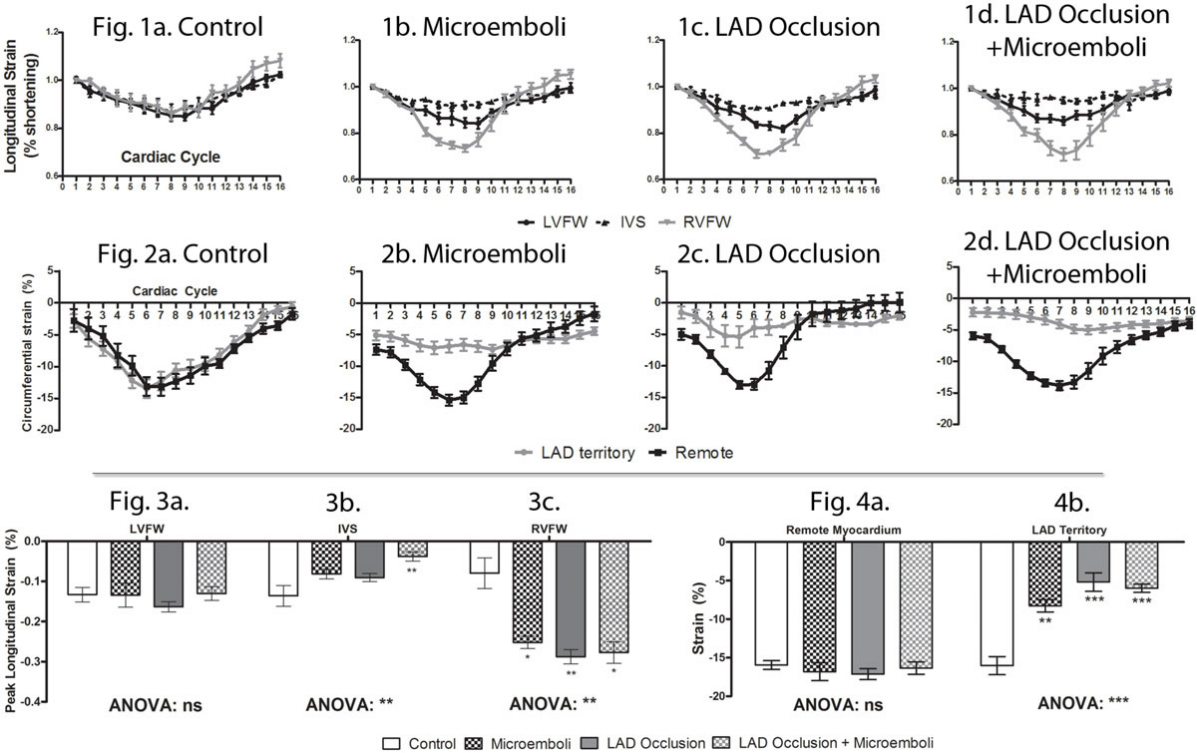
**1 & 2: Phasic longitudinal strain was suppressed after interventions vs. controls**. 3: In contrast, significant increase in peak longitudinal strain was observed in RV after interventions. 4: Severe suppression in peak circumferential strain in IVS after interventions vs. controls.

Control animals showed that there was no difference in longitudinal or circumferential strain between LAD territory (corresponding to IVS) and LV free wall (LVFW) (Figure 1. Parts 1&2). Phasic longitudinal and circumferential strain in LV and RV free walls (RVFW) showed marked differences in temporal strain than controls (Figure 1&2). Coronary interventions caused significant increase in peak longitudinal strain in RVFW (Figure 1 part 3). AMI with microemboli caused severe reduction in IVS longitudinal and circumferential strain vs. controls, although no significant difference was noted between intervention types (Figure 1 part 4). Myocardial damage was significantly different between microembolized, occlusion/reperfusion and AMI with micremboli animals (8.8 ± 0.5% < 12.4 ± 1.2% < 15.7 ± 1.1%).

## Conclusions

MRI showed that segments in pre-existing AMI superimposed with microemboli have impairment in both strain, while segments with single insult showed only circumferential impairment. The interaction between LV and RV after interventions is clearly demonstrated on the RV strain, suggesting that both LV and RV need assessment in AMI.

## Funding

N/A
